# Elements for Successful Advance Care Planning Implementation in Nursing Homes: A Systematic Review

**DOI:** 10.1093/geroni/igaf122.2611

**Published:** 2025-12-31

**Authors:** Peiyuan Zhang, Joan Davitt, Nancy Kusmaul, Paul Sacco, Kathleen Unroe, John Cagle

**Affiliations:** University of Maryland, Baltimore County, Baltimore, Maryland, United States; University of Maryland, Baltimore County, Baltimore, Maryland, United States; University of Maryland, Baltimore County, Baltimore, Maryland, United States; University of Maryland, Baltimore, Maryland, United States; Indiana University School of Medicine, Indianapolis, Indiana, United States; University of Maryland, Batimore, Maryland, United States

## Abstract

Approximately 1.2 million people resided in more than 15,000 nursing homes (NHs) in the U.S., with 30% spending their last days there. Advance care planning (ACP) has well-documented benefits in improving the quality of end-of-life care and thus has significant relevance to NHs. However, the quality of ACP practices varies partly due to a lack of ACP guidelines. This systematic review aimed to identify the key elements for successful ACP in NHs, following the Preferred Reporting Items for Systematic Reviews and Meta-Analysis (PRISMA) guidelines. Three groups of terms representing ACP, implementation processes, and assessment were combined with “AND” and searched in four databases: CINAHL, PsycInfo, SocINDEX, and Medline (Ovid) in June 2024. The search yielded 2,013 studies, of which 83 were reviewed in full, and 19 met the inclusion criteria, validated through independent review and consensus by two researchers. Deductive thematic analysis of the selected studies following Donabedian’s model of quality of care identified two major themes with 11 subthemes: (1) NH structural support for ACP, including policies incorporating ACP into admission, role clarity, and resource availability (e.g., dedicated time and training for ACP, particularly among people with impaired decision-making capacity), and (2) standardized implementation processes, emphasizing resident and family engagement, trust building, discussions of care options and residents’ preferences, documentation of ACP in a standardized place, sharing ACP-related information among an interdisciplinary team, and reviewing ACP regularly. The findings highlight the need for structured support and standardized implementation processes, providing a framework for future NH ACP quality improvement initiatives.

